# Thirty-Days versus Longer Duration of Dual Antiplatelet Treatment after Percutaneous Coronary Interventions with Newer Drug-Eluting Stents: A Systematic Review and Meta-Analysis

**DOI:** 10.3390/life13030666

**Published:** 2023-02-28

**Authors:** Grigorios Tsigkas, Anastasios Apostolos, David-Dimitrios Chlorogiannis, Elena Bousoula, Georgios Vasilagkos, Sotirios Tsalamandris, Ioannis Tsiafoutis, Konstantinos Katsanos, Konstantinos Toutouzas, Adel Aminian, Dimitrios Alexopoulos, Periklis Davlouros

**Affiliations:** 1 Department of Cardiology, University Hospital of Patras, 265 04 Patras, Greece; 2 First Department of Cardiology, University of Athens, Hippokration General Hospital, 115 27 Athens, Greece; 3 Department of Cardiology, General Hospital of Piraeus “Tzaneio”, 185 36 Piraeus, Greece; 4 First Department of Cardiology, Red Cross Hospital, 115 26 Athens, Greece; 5 Department of Radiology, School of Medicine, University of Patras, 265 04 Patras, Greece; 6 Department of Cardiology, Centre Hospitalier Universitaire de Charleroi, 6042 Charleroi, Belgium; 7 Second Department of Cardiology, University of Athens, Attikon University Hospital, 124 62 Athens, Greece

**Keywords:** dual antiplatelet therapy, DAPT, one-month DAPT, percutaneous coronary intervention, PCI, meta-analysis

## Abstract

Abbreviation of the duration of dual antiplatelet therapy (DAPT) (one or three months) has been recently proposed, especially for high bleeding risk patients, after percutaneous coronary intervention (PCI) with drug-eluting stent (DES). Three databases were screened for eligible randomized control trials. The primary endpoint was the incidence of net adverse clinical events (NACE). Secondary endpoints consisted of major adverse cardiovascular events (MACE), all-cause and cardiovascular mortality, myocardial infarction, stroke, stent-thrombosis, repeat revascularization and major bleeding. We included four RCTs with a total of 26,576 patients; 13,282 patients were grouped in 30-days DAPT, while the remaining 13,294 were allocated in a longer period of DAPT. One month of DAPT did not significantly reduce NACE (odds ratio [OR]: 0.87, 95% confidence intervals [Cl]: 0.74–1.03); however, major bleedings were significantly reduced by 22% (OR: 0.78, 95% Cl: 0.65–0.94). Mortality or ischemic events (stroke, myocardial infarction, revascularization and stent thrombosis) were not affected. Thus, 30-days DAPT could be considered as safe and feasible after PCI with DES in selected patients, especially those with high bleeding risk. Forthcoming RCTs could shed light on the optimal duration of DAPT.

## 1. Introduction

Dual antiplatelet therapy (DAPT) remains the cornerstone of medical treatment for patients undergoing percutaneous coronary intervention (PCI) with Drug Eluting Stents (DES) [1,2,3]. Although it prevents ischemic events and stent thrombosis, prolonged DAPT duration is associated with increased bleeding risk. Thus, the shortening of DAPT and the continuation with a single antiplatelet agent, either with aspirin or a P2Y12 inhibitor (clopidogrel, ticagrelor or prasugrel), is an emerging issue in current cardiovascular research.

Recent guidelines have established the six-month duration as the gold standard after coronary stenting for chronic coronary syndromes (CCS) and a twelve-month course for acute coronary syndromes (ACS), while abbreviated regimens could be applied in high- and very high bleeding risk patients [1,3,4,5]. Recent meta-analyses have proven the safety and efficacy of early discontinuation of DAPT and maintenance with a single antiplatelet agent [6,7].

Against this background, recent trials have investigated whether further reduction to thirty-days duration of DAPT is also adequate in patients undergoing PCI. GLOBAL LEADERS was the first trial assessing the safety and efficacy of one-month DAPT and it failed to show superiority against the twelve months-duration of DAPT [8]. In the era of 30-days DAPT, STOPDAPT-2 trial was the first to meet its primary composite (both ischemic and bleeding events) endpoint for both non-inferiority and superiority [9]. Our systematic review and meta-analysis will try to shed light on the risk between ischemic and bleeding risk in patients treated with 30-days DAPT.

## 2. Materials and Methods

Our systematic review and meta-analysis were performed according to the updated Preferred Reporting Items for Systematic reviews and Meta-Analyses (PRISMA) 2020 statement and Cochrane Handbook for systematic review of interventions recommendations [10,11]. Moreover, a PRISMA—P checklist has been completed and is available in Supplementary Materials. Institutional board review approval was not required for a meta-analysis of previous published studies.

### 2.1. Eligibility Criteria and Endpoints

Studies were included in our systematic review and meta-analysis if they met all the following inclusion criteria: (1) randomized-control studies (RCT), (2) DAPT duration ≤ 30 days in the intervention arm, (3) DAPT duration ≥ 90 days in the control group, (4) PCI with DES in all included patients, regardless of the indication, and (5) studies published after 1 January 2015. We decided to set a strict time limit, in order to include only the recent trials using the newer DES and to depict the present clinical status more accurately.

The primary endpoint of the specific project is the incidence of major bleedings, according to Bleeding Academic Research Consortium (BARC) criteria. Secondary endpoints are NACE (Net Adverse Clinical Events), MACE (Major Adverse Cardiovascular Events), all-cause and cardiovascular (CV) mortality, myocardial infarction (MI), strokes, stent thrombosis and need for revascularization. The composite endpoints (NACE and MACE) were applied as defined per included study. The definitions of each endpoint, as they are referred in the included trials, are analyzed in Supplementary Table S1.

### 2.2. Information Sources and Search Strategy

An extensive literature search was conducted in three major electronic databases: Cochrane Central Register of Controlled Trials, Medline and Scopus. The searches were performed on 16 January 2022. In addition, manual search of the reference lists of the included studies was conducted for the identification of other eligible studies. The following keywords were used: “dual antiplatelet therapy”, “dual antiplatelet treatment”, “DAPT”, “percutaneous coronary intervention”, “PCI”, “drug-eluting stents”, and “DES”. The full strategy used in each database is provided comprehensively in Supplementary Table S2.

### 2.3. Selection and Data Collection Process

All the records revealed by the three-databases search were collected and duplicates were removed. Titles, abstracts and keywords of all the articles were evaluated; when the study was considered as a candidate, the full text was assessed. The required data of each study was extracted in a prespecified electronic form. All the previous actions were performed independently by two reviewers; disagreements were solved through discussion with a third author. Extracted data items from each trial were as follows: (i) the report: authors and year of publication; (ii) the study: sample population, inclusion and exclusion criteria, endpoints’ definitions; (iii) the participants: demographical and clinical data; (iv) the procedure: intervention’s indication, stent type; (v) the antiplatelet regimen and duration; and (vi) the clinical outcomes during one-year follow-up.

### 2.4. Risk of Bias Assessment and Statistical Analysis

Risk of bias of included studies was evaluated independently by two authors, using the revised Cochrane ”Risk of Bias” tool for randomized trials (RoB 2.0) [12]. The potent publication bias of each study was assessed by using funnel plots; the sample size of each included trial was plotted against odds ratios (ORs) for each endpoint.

A fixed-effect model (Mantel–Haenszel method) was a priori selected to obtain pooled estimates of each DAPT regimen. The measure of effect was the OR for dichotomous outcomes. When data about an outcome was insufficient, this study was excluded from the analysis about this endpoint. All the analyses were performed in an intention-to-treat basis. To identify study heterogeneity, the statistical inconsistency test [I^2^ = 100% × (Q − df)/Q, where Q = χ^2^ (Cochran’s heterogeneity statistic) and df = its degrees of freedom] was applied. The heterogeneity was classified as follows: low when I^2^ ≤ 25%, moderate when I^2^ ≤ 50% and high if I^2^ > 50% [13]. A two-sided alpha level of 0.05 was considered statistically significant. Review Manager software version 5.4 (Cochrane Collaboration) was used for the previous analyses.

## 3. Results

### 3.1. Search Results

The systematic search of the three databases identified a total of 8681 records. After duplicates were removed, 6086 records were screened through titles, abstracts and keywords. A total of 53 articles was evaluated, after studying the full manuscript. In total, four RCTs were considered as eligible for our systematic review and meta-analysis. PRISMA flowchart is presented in Supplementary Figure S1.

### 3.2. Studies’ Characteristics

The four RCTs included 26,576 patients randomized to either 30-days or standard duration of DAPT (13,282 and 13,294, respectively) [8,9,14,15]. All studies included patients with both acute (ACS) and chronic coronary syndrome (CCS). Management of high bleeding risk patients post bioresorbable polymer-coated Stent implantation with an abbreviated versus prolonged DAPT regimen (MASTER-DAPT) was the only trial, which included exclusively high bleeding risk patients [15]. All studies included one-year clinical follow-up, while GLOBAL LEADERS provided two-years follow-up [8]. All endpoints were evaluated during one-year follow-up. Randomization was performed during index PCI in two studies (GLOBAL LEADERS and One-Month DAPT), and at one month in the others (STOPDAPT-2 and MASTER-DAPT). Standard DAPT regimen consisted of aspirin and clopidogrel in two studies (STOPDAPT-2 and One-Month DAPT), while the others included the rest potent P2Y12 inhibitors. Monotherapy maintenance was performed with different antiplatelet in each study and it was prespecified, except MASTER-DAPT. The characteristics of the included trials are presented in Table 1. Inclusion and exclusion criteria as well as the endpoints of each study are presented in Supplementary Table S3.

### 3.3. Patients’ Characteristics

The baseline demographical, clinical and procedural characteristics are summarized in Table 2. We did not observe noticeable differences among the participants of included studies. Women were under-presented in the included analysis and our meta-analysis, counting less than a third of analyzed patients. Mean age of patients was under 70 years old in the three clinical trials, while MASTER-DAPT included participants with mean age of 76 years old [15]. Most patients suffered from hypertension and dyslipidemia in all the included studies. About one third of included patients had diabetes mellitus under treatment. Notably, about one out of four patients had previously undergone PCI. One-Month DAPT trial did not include patients with ST-Elevation MI and Non-ST-Elevation MI, in contrast with the other studies. Nevertheless, a significant proportion of the analyzed population was catheterized with ACS indication.

### 3.4. Primary Endpoint

**Figure 1:**
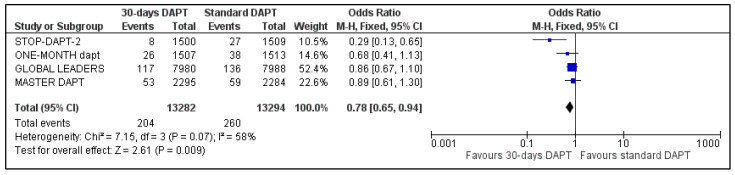
Forest plot showing the impact of 30-days versus >3-months dual antiplatelet treatment on major bleedings, with odds ratio and 95% confidence intervals. CI—confidence interval, DAPT—dual antiplatelet therapy, M–H—Mantel–Haenszel.

Information about major bleedings was available in the whole population of our meta-analysis. Major bleeding was defined when BARC criteria 3–5 were met. Major bleeding occurred in 204 patients in 30-days DAPT and in 260 patients of the comparator arm. Abbreviated regimen resulted in 22% odds ratio reduction of major bleedings [OR: 0.78, 95% Confidence Intervals (CI): 0.65–0.94] (Figure 1). However, heterogeneity was considered as high (I^2^ = 58%, *p* = 0.07).

### 3.5. Secondary Endpoints

A. NACE and MACE

**Figure 2:**
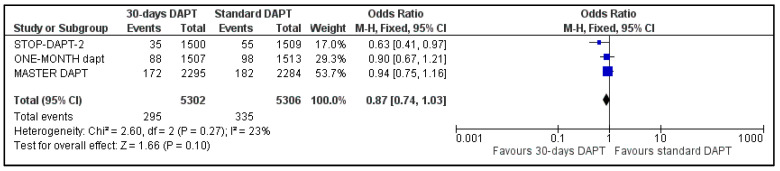
Forest plot showing the impact of 30-days versus >3-months dual antiplatelet treatment on net adverse clinical events, with odds ratio and 95% confidence intervals. CI—confidence interval, DAPT—dual antiplatelet therapy, M–H—Mantel–Haenszel, NACE—net adverse clinical events.

**Figure 3:**
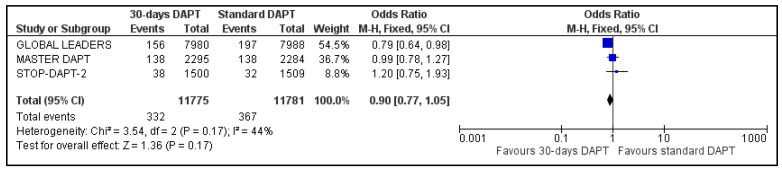
Forest plot showing the impact of 30-days versus >3-months dual antiplatelet treatment on major adverse clinical events, with odds ratio and 95% confidence intervals. CI—confidence interval, DAPT—dual antiplatelet therapy, M–H—Mantel–Haenszel, MACE—major adverse clinical events.

The two composite endpoints of our meta-analysis were available for three trials; data about NACE and MACE were not available for GLOBAL LEADERS and One-Month trial, respectively [8,14]. Pooled analysis showed no significant difference between the two groups, for both NACE (OR: 0.87, 95% CI: 0.74–1.03) (Figure 2) and MACE (OR: 0.90, 95% CI: 0.77–1.05) (Figure 3). Heterogeneity was low (I^2^ = 23%, *p* = 0.27) and moderate (I^2^ = 44%, *p* = 0.17), respectively.

B. All-cause and CV mortality

**Figure 4:**
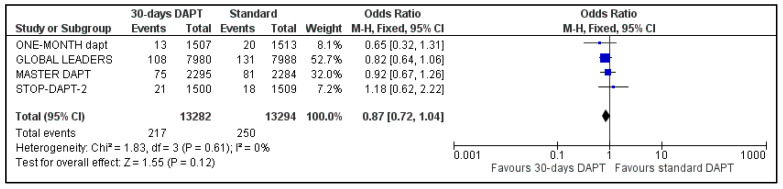
Forest plot showing the impact of 30-days versus >3-months dual antiplatelet treatment on all-cause mortality, with odds ratio and 95% confidence intervals. CI—confidence interval, DAPT—dual antiplatelet therapy, M–H—Mantel–Haenszel.

**Figure 5:**
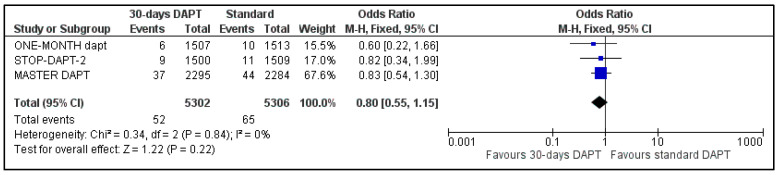
Forest plot showing the impact of 30-days versus >3-months dual antiplatelet treatment on cardiovascular mortality, with odds ratio and 95% confidence intervals. CI—confidence interval, DAPT—dual antiplatelet therapy, M–H—Mantel–Haenszel.

Data regarding all-cause mortality were available for all analyzed patients; nevertheless, GLOBAL LEADERS did not provide results regarding cardiovascular mortality. Regarding all-cause mortality, 217 deaths were observed in 30-days DAPT arm and 250 in the comparator arm (Figure 4). Concerning CV mortality, 52 events were recorded in the intervention and 65 in the control group (Figure 5). No significant difference was estimated regarding all-cause (OR: 0.87 95% CI: 0.72–1.04) and CV mortality (OR: 0.80, 95% CI: 0.55–1.15) with no heterogeneity (I^2^ = 0%, *p* = 0.61 and I^2^ = 0%, *p* = 0.84, respectively).

C. MI and Stroke

**Figure 6:**
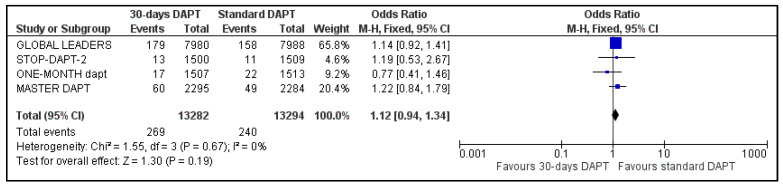
Forest plot showing the impact of 30-days versus >3-months dual antiplatelet treatment on myocardial infarction, with odds ratio and 95% confidence intervals. CI—confidence interval, DAPT—dual antiplatelet therapy, M–H—Mantel–Haenszel.

**Figure 7:**
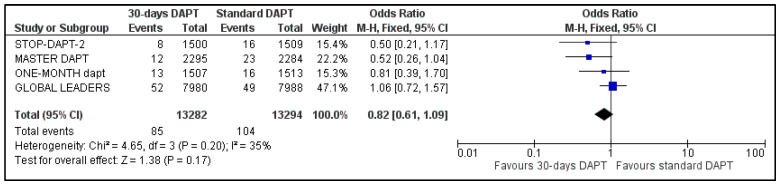
Forest plot showing the impact of 30-days versus >3-months dual antiplatelet treatment on stroke, with odds ratio and 95% confidence intervals. CI—confidence interval, DAPT—dual antiplatelet therapy, M–H—Mantel–Haenszel.

All four included trials provided data regarding MI and stroke. The estimated pooled ORs were 1.12 (95% CI: 0.94–1.34) (Figure 6) and 0.82 (95% CI: 0.61–1.09) (Figure 7), respectively. No heterogeneity was observed for MI (I^2^ = 0%, *p* = 0.67) and moderate heterogeneity was observed for stroke (I^2^ = 35%, *p* = 0.20).

D. Stent Thrombosis and Repeat Revascularization

**Figure 8:**
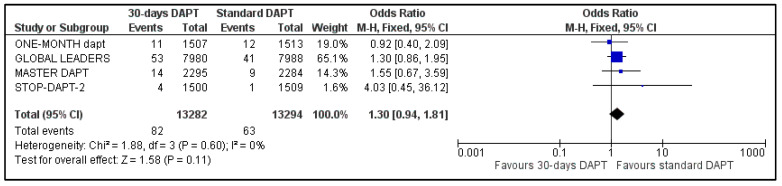
Forest plot showing the impact of 30-days versus >3-months dual antiplatelet treatment on stent thrombosis, with odds ratio and 95% confidence intervals. CI—confidence interval, DAPT—dual antiplatelet therapy, M–H—Mantel–Haenszel.

**Figure 9:**
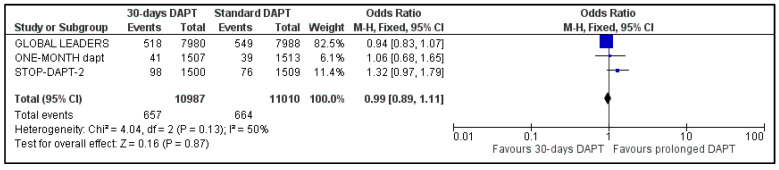
Forest plot showing the impact of 30-days versus >3-months dual antiplatelet treatment for revascularization, with odds ratio and 95% confidence intervals. CI—confidence interval, DAPT—dual antiplatelet therapy, M–H—Mantel–Haenszel.

All the studies reported data about stent stenosis, while MASTER-DAPT did not provide information about revascularization. Pooled analysis showed no significant difference between the two groups, for both stent thrombosis (OR: 1.30, 95% CI: 0.94–1.81) (Figure 8) and urgency for revascularization (OR: 0.99, 95% CI: 0.89–1.11) (Figure 9). Heterogeneity was zero (I^2^ = 0%, *p* = 0.60) and moderate (I^2^ = 50%, *p* = 0.13), respectively.

### 3.6. Risk of Bias Assessment

Risk of bias summary and graph were prepared according RoB 2.0 tool and are presented in Supplementary Figure S2. All included studies were in the lower categories for risk of bias. Publication bias was assessed with funnel plots. Symmetric distribution of the mean effect size was noticed in funnel plots for all outcomes, suggesting low risk of publication bias of the included studies (Supplementary Figure S3).

## 4. Discussion

Major bleeding following PCI remains a major risk factor for increased mortality and rehospitalization [16]. More than 10% of in–hospital post–PCI deaths were related with bleeding complications [17]. Moreover, it is estimated that about 5% of patients undergoing PCI are readmitted due to a hemorrhagic event and they are under higher risk for mortality or MI [18].

Several comorbidities have been related with increased bleeding risk after PCI, such as chronic kidney disease, anemia and heart failure [18,19]. Moreover, increased age, frailty and concomitant oral anticoagulation increase hemorrhagic diathesis [20,21]. Taking these into consideration, several strategies have been attempted to reduce bleedings’ burden. First, several clinical scores predicting the bleeding risk after PCI in patients with CAD have been developed and validated in large cohorts of patients [19]. PRECISE-DAPT score, which was developed by eight RCTs, has been incorporated into 2017 ESC guidelines about DAPT in CAD [22,23]. More recently, ARC-HBR (Academic Research Consortium for High Bleeding Risk), a novel score using 20 risk criteria, has been introduced in clinical practice [24]. The application of such scores could predict the bleeding risk of each patient undergoing PCI and reduce such risk. In addition, the majority of peri-procedural bleeding events occur at the arterial access site [25]. Thus, vascular access through the radial artery, compared to the femoral one, has been associated with significantly less major bleedings and, as a result, with lower mortality rate [26]. As a result, transradial approach has become the gold standard for performing primary and elective PCI, regardless of the indication of catheterization. The use of distal radial artery for coronary catheterization has been associated with even lower incidence of hematomas, comparing to conventional transradial approach [27,28]. Furthermore, ESC guidelines strongly recommend proton pump inhibitor in combination with DAPT [2]. Recently, a large-scale Danish registry revealed that proton pump inhibitors are under-administrated, despite their positive impact on prevention of bleedings [29]. Its use was not associated with increased ischemic complications; nevertheless, omeprazole and esomeprazole should not be administered with clopidogrel, because they interfere with the hepatic activation of clopidogrel and may reduce its efficacy [30].

Moreover, a de–escalation strategy consisting of reducing the dose of P2Y12 inhibitors or shortening of the duration of DAPT, followed by monotherapy with a single antiplatelet agent has been considered as another step in this direction. Current ESC guidelines recommend three-month DAPT in high bleeding risk patients with IIaA and IIaB in CCS and ACS, respectively. Against this context, multiple RCTs and meta-analyses were conducted, in order to investigate whether three-month DAPT is safe and effective in non–high bleeding risk patients undergoing PCI. Indeed, it has been found that this abbreviated regimen is associated with less major bleedings without increasing ischemic complications (MI, stroke, stent thrombosis). Verdoia and her colleagues conducted a meta-analysis, including five RCTs and a total of 30,621 patients and supporting that short-term (<3 months) DAPT significantly reduced major bleedings, without affecting ischemic complications or survival [31]. The previous results were confirmed by another meta-analysis, which included studies with a previous generation of stents [32]. Recently, the results of our meta-analysis focusing on odds of NACEs found that DAPT shorter than three months significantly decreased the NACEs by 17% and the severe bleedings by 29% without increasing ischemic events [33]. Moreover, the trial sequential analysis showed that it was not under-powered and the results could be applied safely. Going a step further, latest ESC guidelines have proposed one-month DAPT with clopidogrel in very high bleeding risk patients with NSTE-ACS or CCS, but with lower evidence and weaker recommendation. Taking that into consideration, numerous RCTs have investigated whether one-month DAPT was safe and feasible. Our meta-analysis confirms the safety and efficacy of shortening DAPT duration to one month [34]. According to our findings, one-month DAPT significantly reduces major bleedings by 21%, without exposing patients to higher ischemic or mortality risk during one-year follow-up. In addition, several meta-analyses have been performed against different backgrounds. Moreover, meta-analyses including only ACS patients or older patients were in line with previous findings [35,36,37].

A special DES has been developed and studied with DAPT for only 30 days, in order to investigate whether it could be considered as a safe option. Resolute Onyx DES was the first FDA-approved for one month DAPT in high bleeding risk patients [38]. The XIENCE 28 study showed that 28 days of DAPT is noninferior comparing to longer DAPT regarding death and myocardial infarction, when a novel cobalt–chromium everolimus-eluting stent is used [39]. The previous results were confirmed for Biofreedom, a polymer-free drug-coated stent; the One-Month trial showed that thirty days DAPT in patients treated with Biofreedom stent was noninferior regarding MACEs, comparing to longer DAPT in other types of stents (Biomatrix or Ultimaster) [14].

Our systematic review and meta-analysis included both high and non–high bleeding risk patients. More specifically, MASTER-DAPT included only patients with high bleeding risk, while the other three included mixed populations. Thus, our findings could be applicable in both populations; nevertheless, the theoretical benefit would be greater in those with high or very high bleeding risk. Montalto et al. showed that abbreviated DAPT is beneficial in patients under oral anticoagulation, while Costa et al. supported that a 1- or 3-month DAPT regimen was related with reduced bleedings and cardiovascular mortality, without increasing ischemic events [40,41].

After DAPT discontinuation, antiplatelet agent selection remains under investigation. Each included trial has followed a different strategy; ticagrelor, clopidogrel and aspirin were administered in GLOBAL LEADERS, STOP-DAPT-2 and One-Month DAPT, respectively. In MASTER-DAPT, antiplatelet selection was an investigators’ decision and clopidogrel was used in 53.9% of patients included in shortened DAPT. Recently, HOST-EXAM supported that chronic administration of clopidogrel was superior comparing to aspirin regarding a composite outcome (all-cause mortality, non-fatal MI, stroke, hospitalization due to ACS and BARC bleeding type 3 or greater) at two-year evaluation [42]. These results were confirmed by the extensive follow-up of HOST exam, five years after the randomization [43]. A recent network meta-analysis supported that P2Y12 inhibitor monotherapy after DAPT discontinuation in patients treated with PCI was superior to aspirin regarding MI incidence, without affecting major bleeding [44]. Current literature remains inadequate and further studies are required for solving this dilemma. Until then, a personalized approach, based on patients’ risk factors for thrombotic and hemorrhagic complications, is necessary.

All studies included patients in either stable or acute event phases. Although the number of patients with ACS was smaller, it remained a significant proportion of the total population (Table 2). Thus, findings were consistent for all-comers, regardless of the procedures’ indication. Keeping in mind that patients with ACS are under higher risk for recurrent MI or stent thrombosis, shortening of DAPT to one month should be performed with more caution. Large-scale RCTs with ACS patients are required for demonstrating the safety and feasibility of 30-days DAPT.

Considering that abbreviated (<6 months) DAPT has not been established as gold standard after PCI with newer generation of DES, it is very early to compare one- versus three-months DAPT. Subanalyses of the existing meta-analyses did not discover any difference between these two time frames’ regimens [32]. However, stents’ evolution, intravascular imaging progress and interventional techniques’ development will reduce further ischemic complications (MI, stent thrombosis and revascularization) and trials studying 30- with 90-days DAPT will try to resolve this dilemma [45,46].

The latest research supporting the shortening of DAPT gains more ground in the era of newer stents and improved interventional techniques. Longer duration of DAPT may be indicated only in specific high-ischemic risk, such as prior stent thrombosis, chronic kidney disease, diabetes mellitus, last patent coronary artery and ST-Elevation myocardial infarction [5,47].

### Limitations

Our systematic review and meta-analysis present a number of limitations that should be addressed. Firstly, it is a study-level meta-analysis; the absence of patient-level data did not allow us to estimate the pooled impact of baseline characteristics to outcomes. Secondly, composite outcomes (NACE and MACE) are defined differently among the included trials; a per-trial definition approach was followed. However, a universal definition for such endpoints would be useful for the better interpretation and evaluation of published data. Thirdly, a small number of studies met the inclusion criteria, and a small number of patients were analyzed. Nevertheless, a sufficient number of patients participated fully, so as to draw interesting conclusions. Fourthly, high heterogeneity was observed in some analyses; thus, each outcome should be evaluated with caution.

## 5. Conclusions

Major bleeding remains a devastating complication following PCI. Numerous approaches have been developed for eliminating bleedings. Our systematic review and meta-analysis showed that a 30-days DAPT reduces major bleeding without increasing ischemic events or mortality rate. Thus, shortening DAPT duration to one month could be considered as a safe and effective preventive measure. More RCTs are required for supporting the results of our systematic review and meta-analysis.

## Figures and Tables

**Table 1:** Study Characteristics.

No	Trial	Year	Duration	Follow-Up	Time of Randomization	Very-Short DAPT	Very-Short DAPT	Very-Short DAPT	Standard DAPT Regimen	Standard DAPT Regimen	Indication for PCI	Stent Used
						Regimen	Duration	Continuation with	Regimen	Duration		
1	GLOBAL LEADERS [8]	2018	2013−2015	2-years	At index PCI	ASA 75–100 mg qd + Ticagrelor 90 bid	1 month	Ticagrelor 90 mg bid	ASA 75–100 mg qd + Ticagrelor 90 bid/Clopidogrel 75 mg qd	12 months	ACS/CCS	Biolimus A9-eluting stent
2	STOPDAPT-2 [9]	2019	2015−2017	1-year	At 1 month	ASA 81–100 mg qd + Clopidogrel 75 mg qd or Prasugrel 3.75 qd	1 month	Clopidogrel 75 mg qd	ASA 81–100 mg qd + Clopidogrel 75 mg qd	12 months	ACS/CCS	cobalt-chromium everolimus-eluting stent
3	One-Month DAPT [14]	2021	2015−2019	1-year	At index PCI	ASA 100 mg qd+ Clopidogrel 75 mg qd	1 month	ASA 100 mg qd	ASA 100 mg qd+ Clopidogrel 75 mg qd	6–12 months	ACS/CCS	polymer-free drug-coated stent or biodegradable-polymer drug-eluting stent
4	MASTER-DAPT [15]	2021	2017−2019	1-year	At 1 month	ASA + Clopidogrel or Ticagrelor or Prasugrel	1 month	NA	ASA + Clopidogrel or Ticagrelor or Prasugrel	3–12 months	ACS/CCS	bioresorbable polymer-coated stent

ACS—Acute Coronary Syndrome; ASA—Acetylsalicylic Acid, CCS—Chronic Coronary Syndrome, PCI—Percutaneous Coronary Intervention.

**Table 2:** Patients’ Characteristics.

	GLOBAL LEADERS [8]		STOPDAPT-2 [9]		One-Month DAPT [14]		MASTER DAPT [15]	
	Short	Standard	Short	Standard	Short	Standard	Short	Standard
Patients (n)	7980	7988	1500	1509	1507	1513	2295	2284
Age (years)	64.5	64.6	68.1	69.1	67	67	76.1	76
Female (%)	23.4	23.1	21.1	23.5	31	31	30.7	30.8
BMI (kg/m^2^)	28.2	28.2	24.4	24.2	24.7	24.7	27.3	27.4
Smoking (%)	25.9	26.3	26.6	20.6	17	16	38.2	37.5
HTN (%)	74	73.3	73.7	74	67	66	76.9	78.2
DM (%)	25.7	24.9	39	38	37	38	32.9	34.3
Dyslipidemia (%)	69.3	70	74.4	74.8	81	82	67.2	68.1
PAD (%)	6	6.7	6.4	6.6	NA	NA	10.6	10.6
CKD (%)	13.9	13.5	5.5	5.6	13	14	18.2	20.1
Previous PCI (%)	32.7	32.7	33.5	35.1	16	18	25.9	26
ACS (%)	47	46.8	37.7	38.6	38	41	49.1	47.4
STEMI (%)	13.3	12.9	19.4	17.9	NA	NA	11.9	11.6
NSTEMI (%)	21.1	21.1	5.4	6.6	NA	NA	25.9	24.4
UA (%)	12.6	12.7	12.9	14.2	35	38	11.3	11.4
CCS (%)	53	53.2	62.3	61.4	62	59	40.2	40.6
No. of stents (n)	NA	NA	1.3	1.3	1.3	1.3	1.47	1.76
Total Length of stents (mm)	NA	NA	30.3	30.5	31	31	39.3	39.7

ACS—Acute Coronary Syndrome, BMI—Body Mass Index, CCS—Chronic Coronary Syndrome, CKD—Chronic Kidney Disease, DM—Diabetes Mellitus, HTN—Hypertension, NSTEMI—Non-ST elevation myocardial infarction; PAD—Peripheric Arterial Disease, STEMI—ST-elevation myocardial infarction, UA—Unstable Angina.

## Data Availability

Data is contained within the article.

## Supplementary Materials

The following supporting information can be downloaded at: https://www.mdpi.com/article/10.3390/life13030666/s1. Figure S1: PRISMA 2020 flow diagram for new-systematic reviews and meta-analyses; Figure S2: Evaluation of risk of bias of each RCT according to the Cochrane Collaboration Tool.; Figure S3A–I: Funnel plots for endpoints; Table S1: Definitions for each endpoint; Table S2: Search Strategy; Table S3: Inclusion, Exclusion Criteria and Endpoints of Each Included Trial.
