# Supplementary material for: Thirty-Days versus Longer Duration of Dual Antiplatelet Treatment after Percutaneous Coronary Interventions with Newer Drug-Eluting Stents: A Systematic Review and Meta-Analysis
Source: Life (Basel). 2023 Feb 28;13(3):666. doi: 10.3390/life13030666 (PMC10056726; doi:10.3390/life13030666)
Supplement: Supplementary file 1 [file life-13-00666-s001.zip › Supplementary Material for 30.pdf]

SUPPLEMENTARY MATERIAL

Supplementary Figure S1: PRISMA 2020 flow diagram for new-systematic reviews and meta-analyses

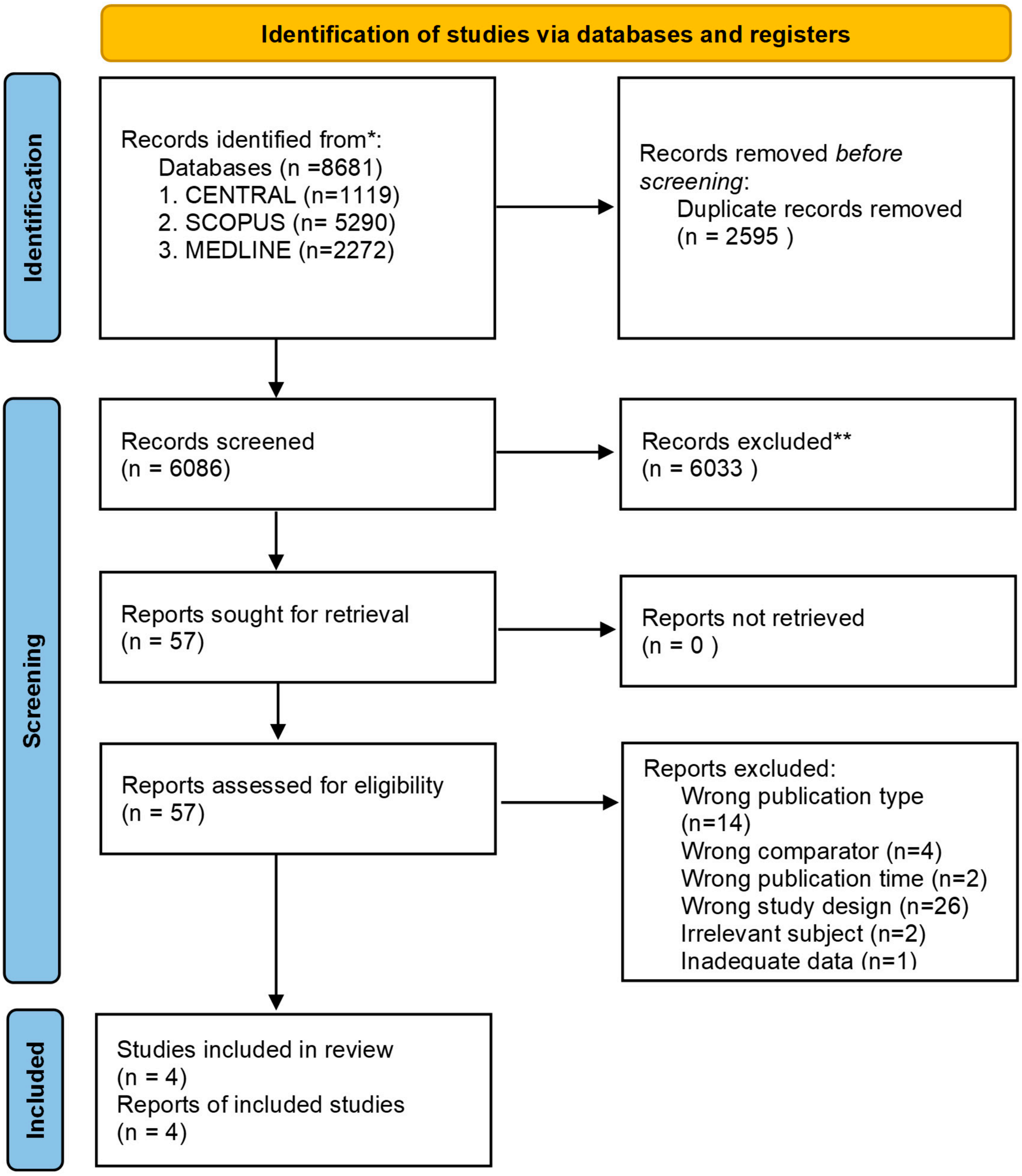

Supplementary Figure S2: Evaluation of risk of bias of each RCT according to the Cochrane Collaboration Tool.

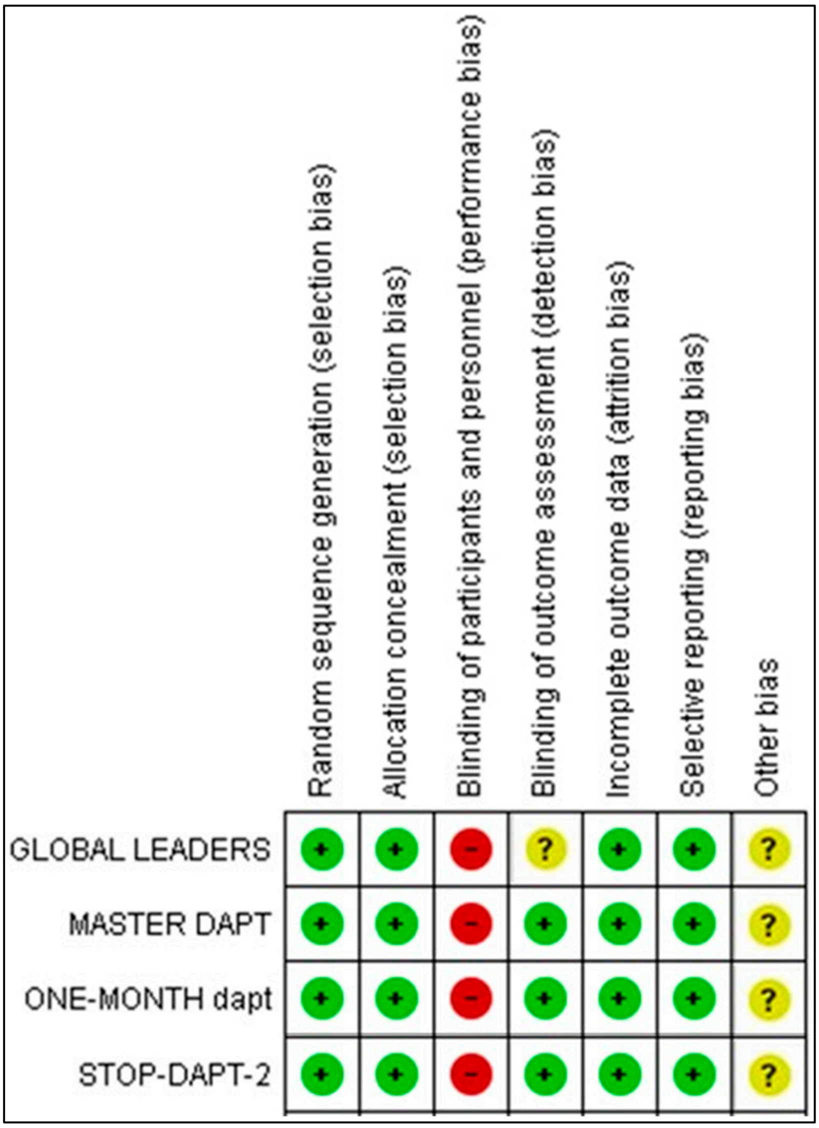

Supplementary Figure S3A: Funnel plot of major bleedings

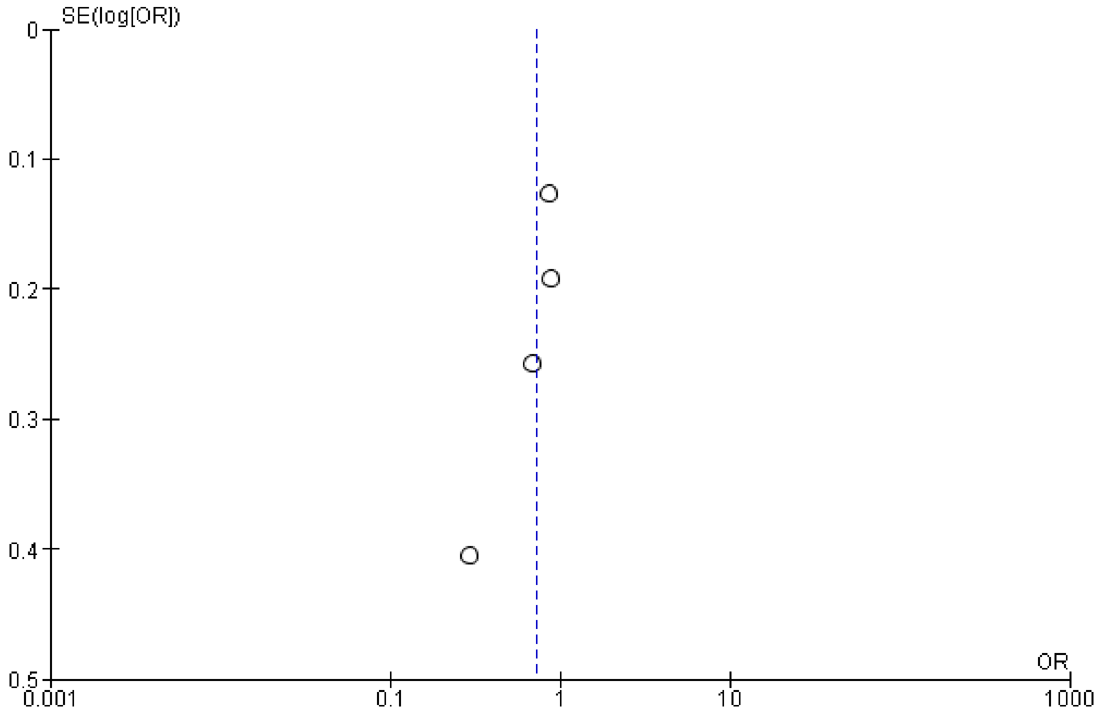

Supplementary Figure S3B: Funnel plot for NACE

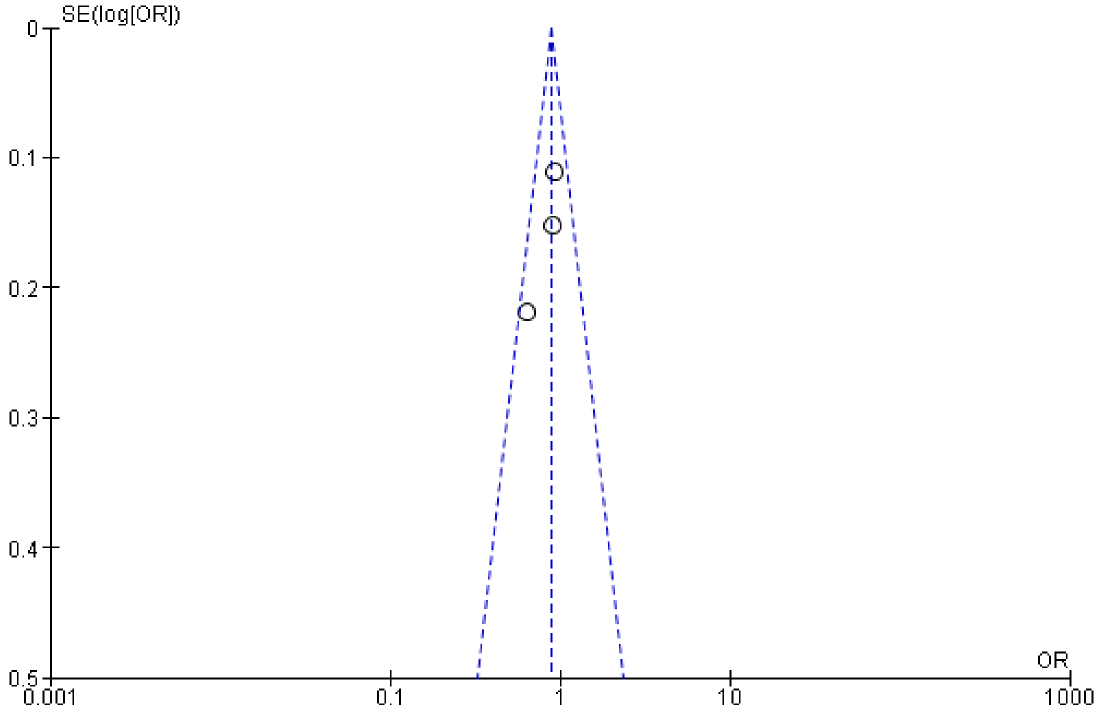

Supplementary Figure S3C: Funnel plot for MACE

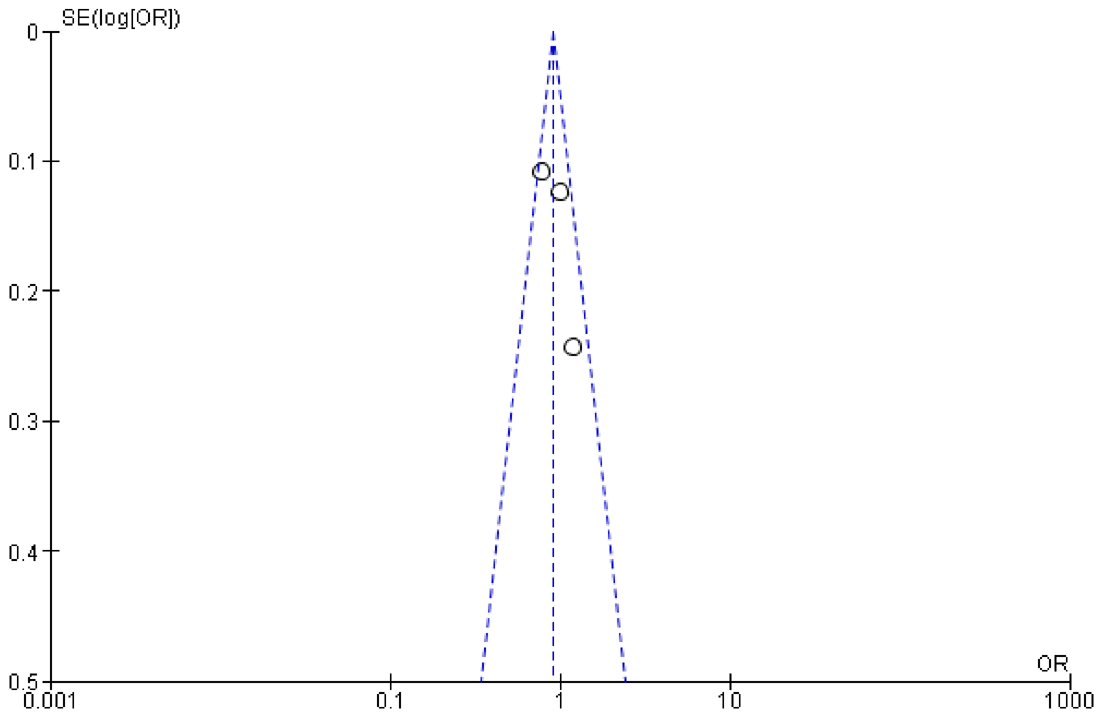

Supplementary Figure S3D: Funnel plot for all-cause mortality

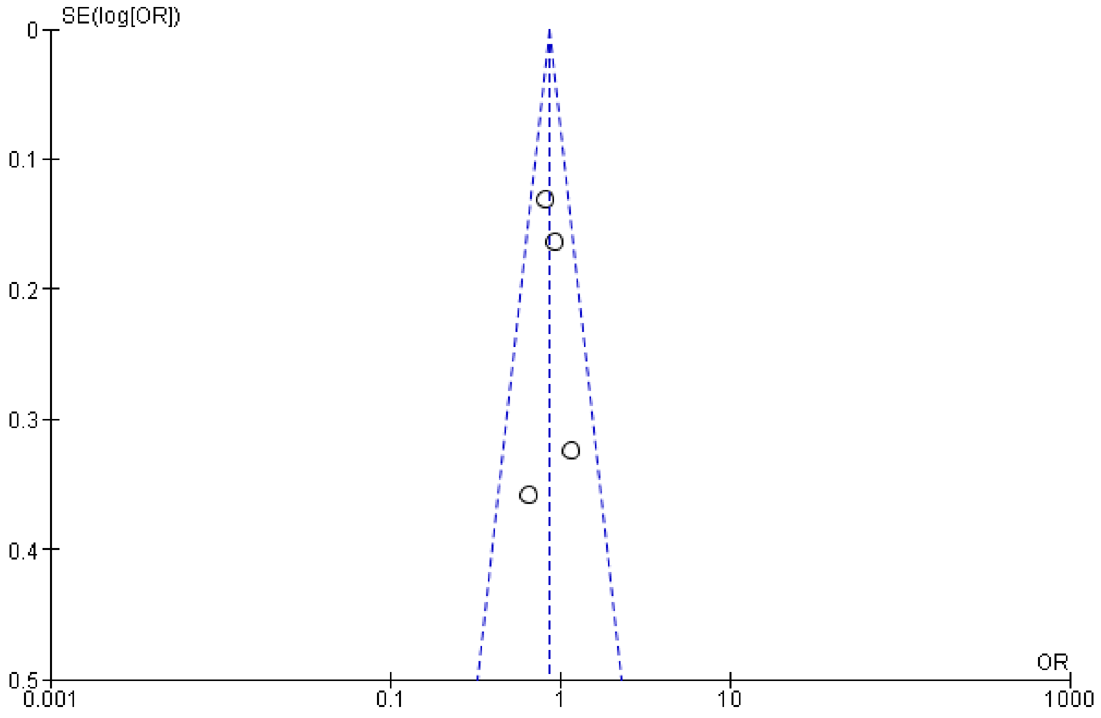

Supplementary Figure S3E: Funnel plot for cardiovascular mortality

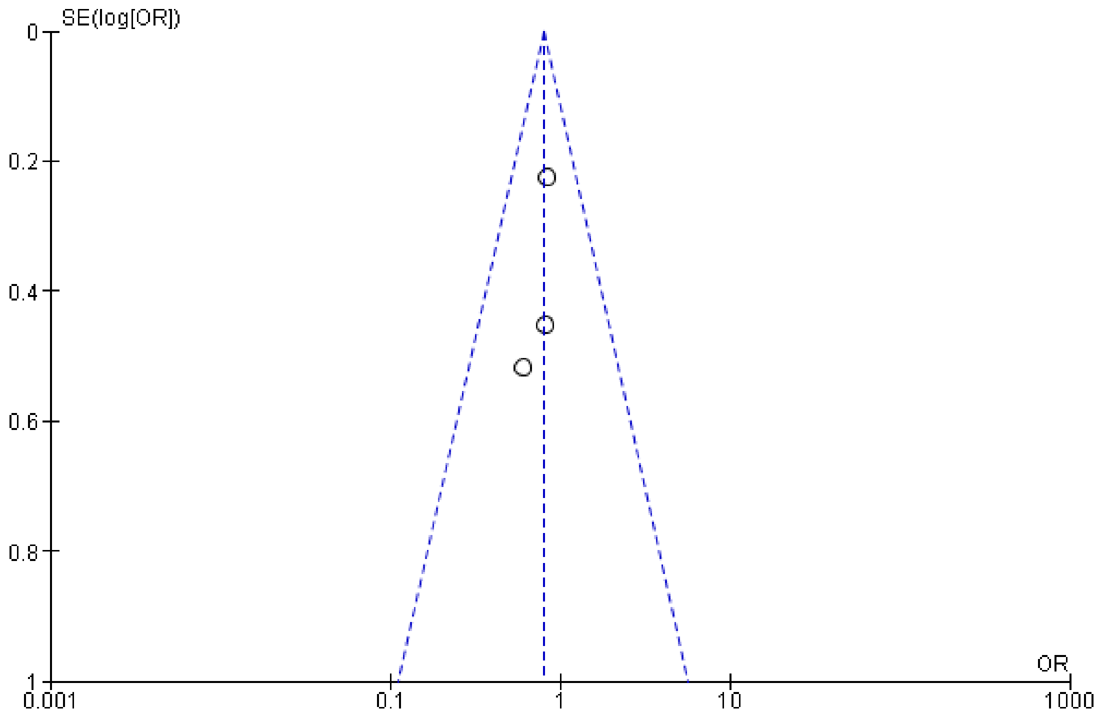

Supplementary Figure S3F: Funnel plot for myocardial infraction.

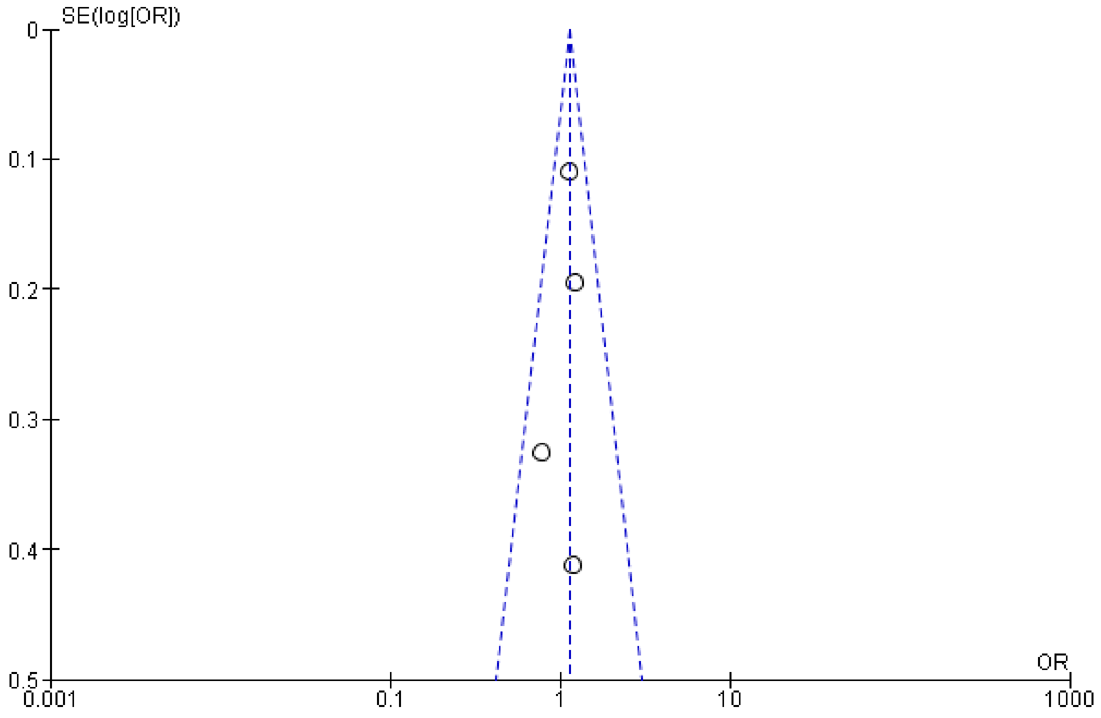

Supplementary Figure S3G: Funnel plot for stroke.

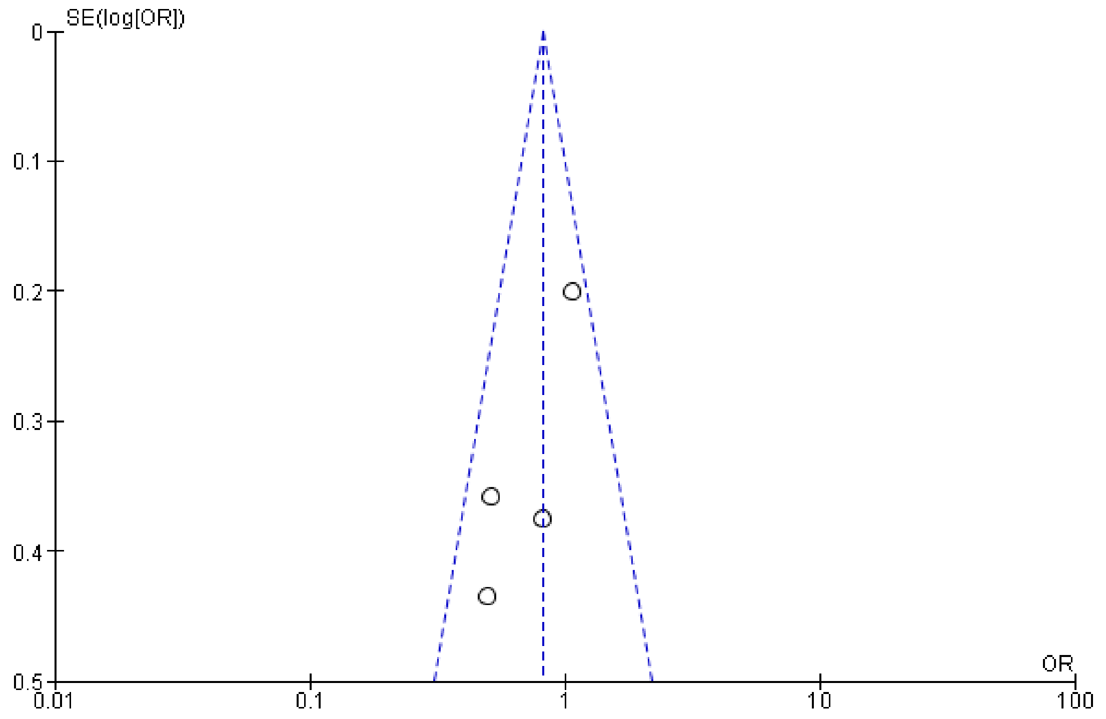

Supplementary Figure S3H: Funnel plot for stent-thrombosis

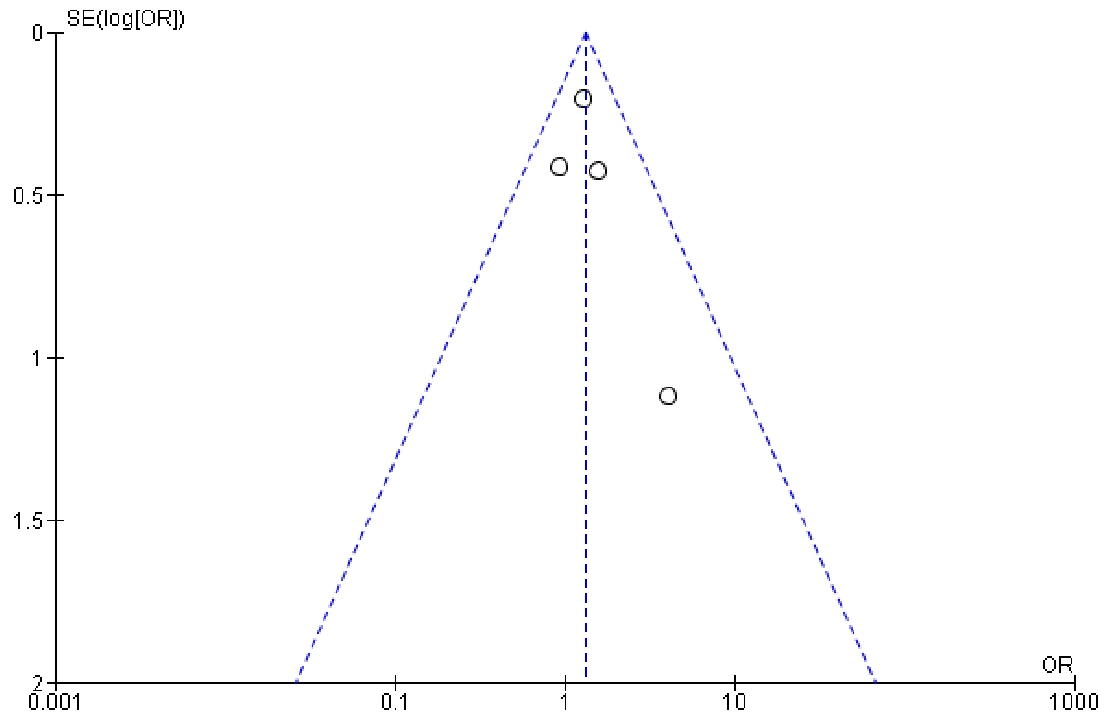

Supplementary Figure S3I: Funnel plot for revascularization

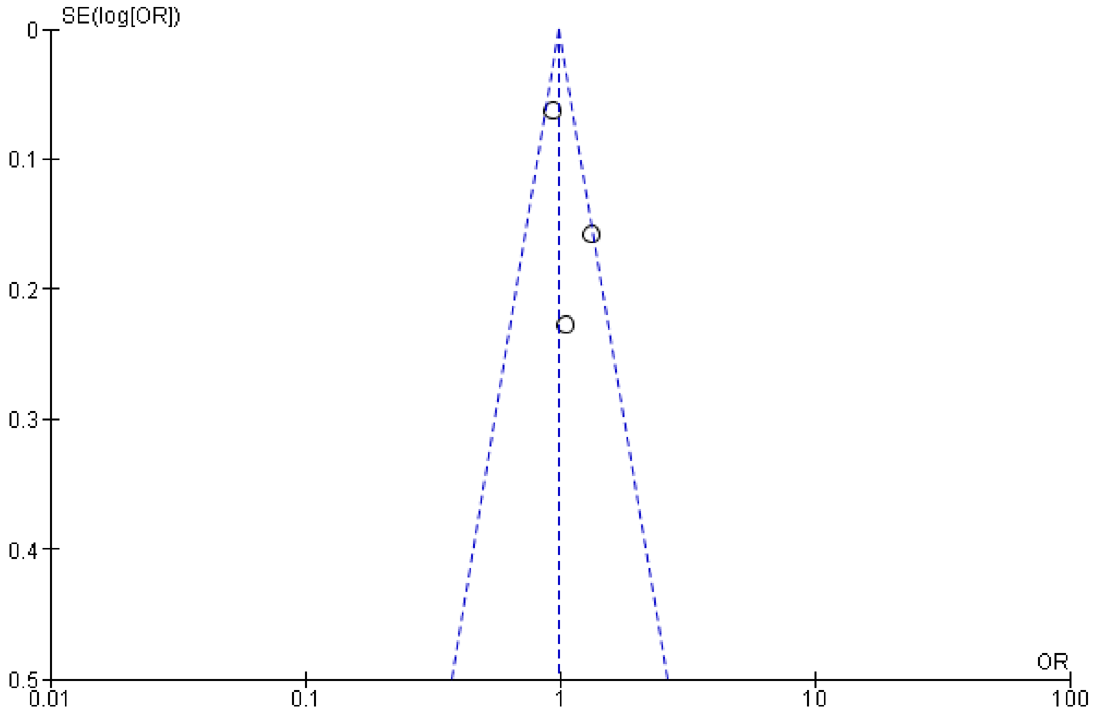

Supplementary Table S1: DEFINITIONS FOR EACH ENDPOINT						
No	Trial	NACE	MACE	Major Bleeding	Revascularization	Stent Thrombosis
1	GLOBAL LEADERS(13)	NA	All-cause mortality, stroke or new Q-wave MI	BARC 3-5	Any revascularization	Definite
2	STOPDAPT-2(15)	Cardiovascular death, MI, definite stent thrombosis, ischemic or hemorrhagic stroke, or TIMI major or minor bleeding.	Cardiovascular death, MI, definite stent thrombosis, or ischemic or hemorrhagic stroke	BARC 3-5	Any coronary revascularization	Definite or Probable
3	One-Month DAPT(19)	cardiac death, nonfatal myocardial infarction, target vessel revascularization, stroke, or major bleeding	NA	BARC 3-5	TVR or target lesion revascularization	Acute, subacute or late and definite, probable or possible

4	MASTER-DAPT(20)	All-cause mortality, MI, stroke cause, myocardial infarction, stroke, or major bleeding, defined as BARC type 3 or 5	All-cause mortality, MI or stroke	BARC 3-5	NA	Definite or probable
BARC; Bleeding Academic Research Consortium, MACE; Major Cardiovascular Events, MI; Myocardial Infraction, NA; Not Available, NACE; Net Adverse Clinical Events, TVR; Target Vessel Revascularization						

Supplementary Table S2: SEARCH STRATEGY		
**Database**	**Search Strategy**	**Results**
Cochrane Central Register of Controlled Trials (CENTRAL)	#1 ("dual antiplatelet therapy"):ti,ab,kw OR ("dual antiplatelet treatment"):ti,ab,kw OR (DAPT):ti,ab,kw	1119 results
	#2 ("percutaneous coronary intervention"):ti,ab,kw OR ("percutaneous coronary angioplasty"):ti,ab,kw OR ("percutaneous coronary revascularisation"):ti,ab,kw OR (stent):ti,ab,kw OR ("DES"):ti,ab,kw	
	#3 #1 AND #2 with Cochrane Library publication date from Jan 2015 to present, in Trials	
	TITLE-ABS-KEY ("Dual antiplatelet therapy" OR "dual antiplatelet treatment" OR dapt) AND TITLE-ABS-KEY ("percutaneous coronary intervention" OR pci OR stent OR des) AND PUBYEAR = 2015	

Scopus	TITLE-ABS-KEY ("Dual antiplatelet therapy" OR "dual antiplatelet treatment" OR dapt) AND TITLE-ABS-KEY ("percutaneous coronary intervention" OR pci OR stent OR des) AND PUBYEAR = 2016	5290 results
	TITLE-ABS-KEY ("Dual antiplatelet therapy" OR "dual antiplatelet treatment" OR dapt) AND TITLE-ABS-KEY ("percutaneous coronary intervention" OR pci OR stent OR des) AND PUBYEAR = 2017	
	TITLE-ABS-KEY ("Dual antiplatelet therapy" OR "dual antiplatelet treatment" OR dapt) AND TITLE-ABS-KEY ("percutaneous coronary intervention" OR pci OR stent OR des) AND PUBYEAR = 2018	
	TITLE-ABS-KEY ("Dual antiplatelet therapy" OR "dual antiplatelet treatment" OR dapt) AND TITLE-ABS-KEY ("percutaneous coronary intervention" OR pci OR stent OR des) AND PUBYEAR = 2019	

	TITLE-ABS-KEY ("Dual antiplatelet therapy" OR "dual antiplatelet treatment" OR dapt) AND TITLE-ABS-KEY ("percutaneous coronary intervention" OR pci OR stent OR des) AND PUBYEAR = 2020	
	TITLE-ABS-KEY ("Dual antiplatelet therapy" OR "dual antiplatelet treatment" OR dapt) AND TITLE-ABS-KEY ("percutaneous coronary intervention" OR pci OR stent OR des) AND PUBYEAR = 2021	
	TITLE-ABS-KEY ("Dual antiplatelet therapy" OR "dual antiplatelet treatment" OR dapt) AND TITLE-ABS-KEY ("percutaneous coronary intervention" OR pci OR stent OR des) AND PUBYEAR = 2022	
MEDLINE	(((("dual antiplatelet therapy"[Title/Abstract]) OR ("dual antiplatelet treatment"[Title/Abstract])) OR ("dapt"[Title/Abstract])) AND (((("percutaneous coronary intervention"[Title/Abstract]) OR ("pci"[Title/Abstract])) OR ("stent"[Title/Abstract])) OR ("des"[Title/Abstract]))) AND (("2015/01/01"[Date - Publication] : "3000"[Date - Publication]))	2272 results

Supplementary Table S3: INCLUSION, EXCLUSION CRITERIA AND ENDPOINTS OF EACH INCLUDED TRIAL					
	**Trial**	**Inclusion Criteria**	**Exclusion Criteria**	**Primary Endpoint**	**Secondary Endpoints**
1	GLOBAL LEADERS(13)	Age ≥18 years, clinical indication for PCI, presence of one or more coronary artery stenosis of ≥50% in a native coronary artery or in a saphenous venous or arterial bypass conduit suitable for coronary stent implantation in a vessel with a reference vessel diameter of at least 2.25 mm	Intolerance to aspirin, P2Y12 inhibitors, bivalirudin, stainless steel or biolimus, known intake of a strong cytochrome P3A4 inhibitor as co-administration may lead to a substantial increase in exposure to ticagrelor, use of fibrinolytic therapy within 24 h of PCI, planned CABG as a staged procedure (hybrid) within 12 months of the index procedure, planned surgery within 12 months of PCI unless DAPT is maintained throughout the peri-surgical period, need for oral anti-coagulation therapy, PCI for a	Composite of all-cause mortality or non-fatal centrally adjudicated MI	BARC (3 or 5), individual components of the primary endpoint; a composite endpoint of all-cause death, MI, or stroke, TVR or any revascularization; and definite stent thrombosis

			priori known stent thrombosis, overt major bleeding, history of intracranial hemorrhage, stroke from ischemic or unknown cause within last 30 days		
2	STOPDAPT-2(15)	Patients who have undergone PCI with the everolimuseluting cobalt-chromium stent (CoCr-EES, XienceTM) and have not experienced major complications (death, MI, stroke, or major bleeding) during hospital stay for treatment	DES other than Xience implanted in PCI performed at the time of enrollment, need for oral anticoagulation or antiplatelet therapy other than aspirin and P2Y12 inhibitors, history of intracranial bleeding, and known intolerance to clopidogrel	Composite of cardiovascular death, MI, ischemic or hemorrhagic stroke, definite stent thrombosis, or major or minor bleeding at 12-month	Cardiovascular death, MI, ischemic or hemorrhagic stroke, or definite stent thrombosis, or major or minor bleeding

3	One-Month DAPT(19)	Age ≥19, ischemic heart disease who are considered for treatment with PCI<br>And significant coronary de novo lesion	MI, complex lesion morphologies such as aorto-ostial, unprotected left main, chronic total occlusion, graft, thrombosis, heavy calcified or extremely tortuous lesion, need to use of DAPT more than 1 month because of other medical conditions, cardiogenic shock or experience of cardiopulmonary resuscitation, contraindication or hypersensitivity to Biolimus A9 or Sirolimus, stainless steel, heparin, antiplatelet agents or contrast media, history of documented prior cerebrovascular attack within 6 months, treated with any stent within 3 months, reference vessel diameter <2.25 mm or >4.0 mm, pregnant women or women with potential childbearing,	Composite of cardiac death, nonfatal myocardial infarction, target-vessel revascularization, cerebrovascular accident and major bleeding at 12 months	All-cause death, cardiac death, non-fatal MI, TVR, stent thrombosis, stroke and major bleeding

			inability to follow the patient over the period of 1 year after enrollment, as assessed by the investigator, inability to understand or read the informed content		
4	MASTER-DAPT(20)	Age ≥18, at least one high bleeding risk criterion, all coronary lesions successfully treated with Ultimaster stent, free of any flow-limiting angiographic complications that required prolonged DAPT duration based on operator's decision, all stages of PCI were complete and no further PCI was planned, at least one high bleeding risk criterion	treated with stent other than Ultimaster stent within 6 months prior to index PCI, treated for in-stent restenosis or stent thrombosis at index PCI or within 6 months before, treated with a bioresorbable scaffold at any time prior to index procedure, incapable of providing written informed consent, under judicial protection, tutorship or curatorship, unable to understand and follow study-related instructions or unable to comply with study protocol, active bleeding	a composite of death from any cause, myocardial infarction, stroke, or major bleeding, a composite of death from any cause,	A composite of death from cardiovascular causes, myocardial infarction, or stroke; all-cause death; stent thrombosis;

		(listed above) or on the basis of post-PCI actionable nonaccess-site related bleeding episode, uneventful 30-day clinical course (i.e. freedom from any new episode of ACS, symptomatic restenosis, stent thrombosis, stroke, any revascularization requiring prolonged DAPT) If not on OAC: a) Patient was on DAPT regimen of aspirin and a P2Y12 inhibitor; b) Patient with one type of P2Y12 inhibitor for at least 7 days 4. If on OAC: a) Patient was on the same	requiring medical attention BARC ≥2 on randomization visit, life expectancy less than 1 year, known hypersensitivity or allergy to aspirin, clopidogrel, ticagrelor, prasugrel, cobalt chromium or sirolimus, any planned and anticipated PCI, participation in another trial, pregnant or breastfeeding women	myocardial infarction, or stroke	

		type of OAC for at least 7 days; b) Patient was on clopidogrel for at least 7 days			
ACS; Acute Coronary Syndrome, BARC; Bleeding Academic Research Consortium, CABG; Coronary Artery Bypass Graft, CAD; Coronary Artery Disease, CKD; Chronic Kidney Disease, DAPT; Dual Antiplatelet treatment, DES; Drug Eluting Stent, DM; Diabetes Mellitus, LAD; Left Anterior Descending, MI; Myocardial Infraction, PCI; Percutaneous Coronary Intervention, TVR; Target Vessel Revascularization					
